# Body composition outcomes of Healthy Fit and the role of acculturation among low-income Hispanics on the US-Mexico border

**DOI:** 10.1186/s12889-021-11015-0

**Published:** 2021-05-25

**Authors:** Diane I. Lopez, Lauren Chacon, Denise Vasquez, Louis D. Brown

**Affiliations:** 1 The University of Texas Health Science Center at Houston, School of Public Health in El Paso, 5130 Gateway East Blvd., El Paso, TX 79905 USA; 2 The University of Texas at El Paso, El Paso, USA

**Keywords:** Obesity, Hispanics, Immigrants, Physical activity, Acculturation

## Abstract

**Background:**

Hispanic immigrants continue to experience higher rates of overweight and obesity compared to their non-Hispanic counterparts. Acculturation may contribute to unhealthy weight gain among immigrant populations by shifting dietary patterns from high fruit and vegetable consumption to unhealthier high fat diets. Healthy Fit, a culturally tailored community health worker (CHW) intervention, aims to reduce obesity related outcomes by providing physical activity and nutrition education and resources in a low-income Hispanic population. This study aims to evaluate outcomes of Healthy Fit participants and examine changes in body composition in relation to level of acculturation at baseline and follow-up.

**Method:**

In this longitudinal observational study, CHWs recruited 514 participants from community events and agencies serving low-income Hispanic populations in El Paso, Texas from 2015 to 2016. Following an in-person health screening, eligible participants received nutrition and physical activity education guided by fotonovelas, comic-like educational books. Telephone follow-ups made at 1, 3, and 6 months by CHWs encouraged follow-through on referrals. 288 participants completed the screening again during the 12-month follow-up.

**Results:**

The sample was predominantly Hispanic (96%), female (82%), uninsured (79%), had a household income of less than $19,999 (70%), foreign-born (79%), preferred Spanish (86%) and few rated themselves as good or excellent for English proficiency (27%). Overall, Healthy Fit participants significantly improved (i.e., decreased) BFP by 0.71% (*t* = 2.47, *p* = 0.01) but not BMI (*b* = .01, *t* = − 0.14, *p* = .89). Contrary to expectations, acculturation was not associated with lower BMI (*b* = 0.09, *p* = 0.84) or BFP (*b* = 0.13, *p* = 0.85) at baseline. However, acculturation predicted changes in both BMI (*b* = 0.30, *p* = 0.03) and BFP (*b* = 1.33, *p* = 0 .01) from baseline to follow-up. Specifically, the low acculturation group improved in body composition measures over time and the high acculturation group did not improve in either measure.

**Conclusion:**

Findings suggest Healthy Fit was most effective among less acculturated individuals. The influence of acculturation on the efficacy of nutrition and exercise interventions suggests that Hispanics should not be treated as a homogenous subgroup.

## Background

Obesity markedly increases risk for cardiovascular diseases, hypertension, type 2 diabetes, stroke, and some type of cancers, costing billions of dollars in healthcare expenses [1]. In 2016, over 70% of adults aged 20 and older in the United States (US) were overweight or obese [2]. The estimated direct medical care cost to the US healthcare system ranges from $147 billion to $210 billion per year for preventive and treatment services, and an additional $4.3 billion is estimated in lost productivity due to job absenteeism [3]. Unfortunately, rates of obesity and overweight are expected to increase, with minority populations disproportionately affected [1]. Of the obese or overweight US adult population, 42% are Hispanic compared to 34.5% for non-Hispanic whites [1].

In 2018, immigrants made up over 13% of the nation’s population, with about half of the immigrant population from Mexico (25%) and other Latin American countries (25%) [4]. This population is less likely to understand English, have lower educational attainment and income and are more likely to be uninsured [5], increasing their risk for adverse health outcomes. The persistent disparity in social determinants is exacerbated by inequities in the physical environment [6]. For example, lower income usually dictates the consumption of cheaper foods, usually containing high levels of fats and sugar, thereby increasing risks for unhealthy weight gain and diabetes [7]. The current food environment is also one that allows for abundant, cheaper food served in large portions, with many fast-food locations concentrated in lower-income and minority communities [6]. Meeting physical activity recommendations is also inversely related to income, that is, lower income communities have the least percentage of people engaging in physical activity [8]. The built environment, such as poor infrastructure or neighborhood safety, may also limit a persons’ ability to engage in physical activity safely and regularly. Adults with limited access to parks or walking trails are two times less likely to meet physical activity guidelines compared to other communities that are more walkable or have recreational facilities [9, 10]. In addition to socioeconomical and environmental factors increasing risk for obesity, Hispanic immigrants experience other barriers to accessing health care and preventive services that could minimize poor health outcomes, such as poor English proficiency and fear of deportation [5].

Despite barriers, several health measures among Hispanic immigrants reflect outcomes contrary to what may be expected considering common socioeconomic characteristics of Hispanic communities. That is, Hispanics tend to have better health outcomes and longer life expectancy compared to their non-Hispanic white counterparts despite having lower income and less access to health care resources [11]. This Hispanic paradox observably diminishes among later generations and as first-generation immigrants become more acculturated to US behaviors and beliefs, such as transitioning to a Western-typical diet [12]. For example, US-born Hispanics have a higher prevalence of obesity, hypertension, smoking, heart disease, and some types of cancer compared to foreign-born Hispanics [13]. These findings may be due to the Healthy Immigrant Effect (HIE), which suggests that new immigrants are in better health due to practicing healthier behaviors in their origin country or by self-selection, where only the healthiest immigrate to find work in the US [14, 15]. Research suggests this health advantage also dissipates with more time living in the US [15]. The nutritional transition theory also explains diminished health outcomes as a result of immigrants transitioning from a diet high in fruit and vegetable intake to a high-calorie, high-fat diet [16].

Acculturation, which is the process of immigrants adopting behaviors and attitudes of the new culture, increases risk for obesity in the US [15]. For example, immigrants who resided in the US for 10 or more years, and were thus more acculturated, had a higher Body Mass Index (BMI) compared to immigrants in the US for less than 10 years [17]. BMI among foreign-born immigrants approached US-born counterparts’ BMI as time living in the US increased [17, 18]. Less acculturated individuals possess several unique characteristics that may influence their response to weight related interventions, including low rates of correct weight perception, weight dissatisfaction, weight loss intention, and weight loss success, along with higher fruit and vegetable consumption [19, 20]. Individuals who accurately perceive their weight as overweight or obese are more likely to pursue and maintain weight loss in comparison to those who do not recognize themselves as such [20, 21]. Correct weight perception is also influenced by a healthcare provider, highlighting the importance of language in being able to effectively navigate the healthcare system, understand health recommendations, and engage in resources [21]. Since less acculturated individuals are less likely to proficiently speak English, language also serves as a factor in the success of weight loss interventions.

Since acculturation influences cognitions and behaviors related to weight, it is important to consider when developing interventions targeting immigrant communities. Cultural tailoring, modifying an intervention to appropriately incorporate elements of a population’s culture, improves the efficacy of behavior change interventions [22]. However, culturally appropriate interventions and preventive programs for Hispanics, specifically with low English proficiency, are lacking [20]. Promising methods of cultural tailoring for Hispanic populations include the use of community health workers (CHWs) and fotonovelas [23]. CHWs are members of the community that have been trained on health education delivery and available community resources. They are successful in reaching underserved communities and ethnic minority groups because they often relate to the lived experience of the target population [24]. CHWs advocate for the community’s health and empower individuals to make healthy behavior changes, in addition to linking disadvantaged communities to resources [24]. Interventions that are tailored to match the cultural identity and language of target communities are more likely to lead to positive health outcomes [22].

Fotonovelas, comic-like educational books, have been used to increase awareness and promote healthy habits in Hispanic populations [23]. This creative narrative approach allows participants to self-identify with characters and is easily understood, allowing participants with limited literacy to effectively engage in the educational material [24]. The fotonovelas used for Healthy Fit are a series of comic-books that were developed with the help of CHWs as part of Project HEART (Health Education Awareness Research Team) study [25]. These fotonovelas follow a Hispanic family, the Ramirez’s, through their struggle with chronic disease management and prevention. The fotonovelas, “An Ounce of Prevention” and “How to Control Your Blood Pressure”, use Hispanic values such as family, respect, and spirituality to promote health behaviors; for example, the use of spirituality to reduce stress for hypertension prevention. CHWs who are less acculturated are better able to explain the Ramirez family and make it relatable to the participant to point out common unhealthy behaviors and the importance of changing these with support from the family. Community intervention programs utilizing CHWs and similar fotonovelas improve access to health care, knowledge, and promote positive health outcomes [24].

Healthy Fit, the intervention examined here, is strengthened by its culturally and linguistically competent use of fotonovelas and low acculturated CHWs. Therefore, we expect Healthy Fit to be more effective at promoting weight loss among Hispanic individuals, specifically those with lower levels of acculturation. The purpose of this study is to evaluate the health outcomes of Healthy Fit participants 12 months after the initial intervention. Results from the 6-month follow-up suggest Healthy Fit improved exercise and nutrition, but did not include measures of body composition [26]. As part of this analysis, we examine changes in body composition in relation to acculturation status.

Based on the previously reviewed acculturation research, our first hypothesis is that more acculturated participants will have a higher body mass index (BMI) and body fat percentage (BFP) at baseline. Secondly, acculturation will significantly predict changes in body composition measures over time. Lastly, among participants, we expect less acculturated participants to experience greater reductions in body composition relative to more acculturated individuals, since Healthy Fit utilizes CHWs with low acculturation to deliver education and fotonovelas designed for less acculturated immigrants.

## Methods

### Participants

The population of interest was low-income, uninsured individuals and Medicaid beneficiaries, 18 years or older. Although insured individuals were not excluded, CHWs recruited during community events that primarily served low-income, uninsured Hispanic populations. Adults of other ethnicities were also included although not particularly targeted. Hispanic ethnicity was self-identified, regardless of language spoken or generation in the US. The only exclusion criterion was for pregnant women due to our interest in tracking body composition changes over time.

CHWs recruited participants by attending large community events and health fairs where multiple programs promoted services in the community. Specifically, 352 (68%) of participants were recruited by CHWs at the Mexican Consulate in collaboration with Ventanilla de Salud (Window of Health) and 58 (11%) at Ayuda, a nonprofit community organization. CHWs promoted the free screening and services, which included a $5 cash incentive for completing the survey and screening. When they showed interest, CHWs explained the consent form, the Healthy Fit program, and the study. CHWs informed potential participants that the program was part of a large study conducted in partnership with the El Paso Department of Public Health. CHWs also explained the body composition measurements that would be taken to inform them of their risk for cardiovascular diseases. After addressing any questions or concerns expressed by the participant, the CHW obtained the individual’s signature on a consent form. After receiving the free screening, education and connection to free services, many participants offered to share the program flyer with family members or friends whom they knew were uninsured or could benefit from the program, which helped to recruit others of similar socioeconomic status. The study protocol for recruitment, consent form, and all project materials were reviewed and approved by the Institutional Review Board at The University of Texas Health Science Center at Houston.

### Data collection-Healthy Fit intervention

This longitudinal observational evaluation of Healthy Fit examines a sample of 514 participants recruited by five CHWs from February 2015 through May 2016. All CHWs were Hispanic and fluent in Spanish. Most participants were recruited by CHWs who lived most of their life in Mexico and strongly preferred Spanish over English. The primary goal of Healthy Fit was to reduce Hispanic health disparities related to obesity, hypertension, cardiovascular disease, and improve access to preventive health services [26]. Healthy Fit consists of an initial 20–45-min health screening conducted by CHWs, followed by referrals to clinical and community resources.

Overweight/obese or hypertensive participants at baseline received heart health fotonovelas and a list of free or donation-based community exercise events, including Zumba dance classes, yoga, and walking groups. Although participants who had a healthy BMI and blood pressure measurements did not receive the fotonovelas with tailored heart health education, they did receive a copy of the list of exercise opportunities. For adults, overweight is considered a BMI of 25–29.9 and obese is a BMI of 30 or greater [1].

As mentioned, the fotonovelas were culturally tailored health education materials used in the Mi Corazón Mi Comunidad (My Heart My Community) curriculum previously tested as part of Project HEART [25, 27]. The fotonovelas, developed at an elementary literacy level, contained information and activities to improve diet and exercise. To facilitate the integration of friendship during exercise, CHWs encouraged participants to attend and invite friends to the community-based exercise events.

CHWs conducted telephone follow-ups at 1, 3, and 6 months to assess and encourage follow through on referrals provided. As barriers to attending exercise classes were mentioned during the initial screening or during follow-up phone calls, CHWs provided encouragement and recommended attending with a friend. At 12-months, CHWs repeated the in-person health screening to assess changes in body composition for all participants, including normal weight participants. The response rate for follow-up at 12-months was 56%, with 288 participants completing the in-person interview and repeat body composition measures.

### Measures

The health-screening instrument included questions on demographics and health status that determined eligibility for referrals. Demographic measures included age, gender, language preference, English proficiency, health insurance coverage, yearly household income range, and level of education. The Healthy Fit survey instrument was adapted from the NIH-funded PhenX Toolkit protocols, where available [28]. When pre-existing Spanish versions of the questions were not available, researchers and CHWs collaborated to translate instruments into Spanish, with back translation into English, to refine accuracy of translations [29].

CHWs used a stadiometer to measure height and a scale with bioelectrical impedance analysis to measure weight and BFP. Due to occasional technical challenges with the biometric impedance scale, BFP was sometimes not collected. Body composition changes were calculated using the baseline and 12-month follow-up measurements.

The variable socioeconomic status (SES) was created by combining z-scores on level of educational attainment and yearly income into one mean score. Following the PhenX protocol, acculturation was measured via an assessment of birthplace (US or foreign-born), number of years living in the US, English proficiency, and language preference, which are widely accepted markers of acculturation [30, 31]. We created an aggregate acculturation score (α = .80) to create a single robust measure by first standardizing each of the four measures of acculturation and then computing their mean. This method reduces measurement error and the number of analyses required, thereby reducing the possibility of Type II error. Acculturation level of CHWs was assessed using the same aggregate score. The acculturation variable was later split by the mean into “high” and “low” groups for follow-up analyses.

### Analysis

We conducted a Pearson correlation to assess associations between all variables: BMI, BFP, acculturation, SES, age, and gender. We also conducted separate multivariate linear regression models to examine if acculturation predicted BMI and BFP at baseline and follow-up. Regressions at follow-up controlled for baseline levels of the dependent variable, thus making acculturation a predictor of change over time [32]. We included age, gender, and SES as covariates in the regression models based on evidence of their impact on body composition [33]. Participants classified as normal BMI and/or BFP were included in all analyses. To test for moderation effects, each covariate was multiplied by the focal predictor, acculturation, to create an interaction term [34]. We tested the linear regression assumptions of normality of residuals, collinearity, homogeneity of variance, and influential observations. To test if the mean differences in body composition between the baseline and follow-up time points was statistically significant, we conducted a paired sample t-test for both BMI and BFP. Finally, as a follow-up analysis based on significant findings from baseline to follow-up, we used independent sample t-tests to determine if differences by acculturation group were significant. All statistical analysis was conducted in Statistical Analysis System (SAS) University Edition.

## Results

The sample characteristics presented in Table 1, *N* = 514 for the entire sample was predominantly Hispanic (96%), female (82%), uninsured (79%), and had a household income of less than $19,999 (70%). In terms of acculturation, most of the sample was foreign-born (79%) with an average of 21 years residing in the US. The majority also preferred speaking and writing in Spanish (86%) and few rated themselves as good or excellent for English proficiency (27%). At baseline, the mean BMI was 30.58 and the mean BFP was 42.02%. The prevalence of obesity was 48.6% (*n* = 250), with an additional 33.3% (*n* = 171) overweight (Table 1).

**Table 1:** Frequency of key sample characteristics (*n* = 514 overall, *n* = 164 for low acculturation, *n* = 94 for high acculturation)

Characteristic	Overall (***n*** = 514) n (%)	Low Acculturation (164) n (%)	High Acculturation (***n*** = 94) n (%)	***p***-value
Gender				0.0128
Male	93 (18.1)	14 (8.5)	18 (19.1)	
Female	421 (81.9)	150 (91.5)	76 (80.9)	
Hispanic Ethnicity				0.7859
Yes	498 (96.8)	156 (95.1)	91 (96.81)	
No	11 (2.1)	6 (3.7)	2 (2.1)	
Missing*	5 (0.1)	2 (1.2)	1 (1.1)	
Income				0.0057
< $19,999	363 (70.6)	121 (73.8)	53 (56.4)	
$20,000 - $29,999	84 (16.3)	29 (17.7)	20 (21.3)	
$30,000 - $39,999	30 (5.8)	7 (4.3)	10 (10.6)	
$40,000+	31 (6.0)	6 (3.6)	10 (10.6)	
Missing*	6 (1.2)	1 (0.6)	1 (1.1)	
Educational Attainment				<.0001
< High School diploma	247 (48.0)	103 (62.8)	14 (14.9)	
High School Grad or GED	115 (22.4)	35 (21.3)	35 (37.2)	
Some college	94 (18.3)	16 (9.8)	30 (31.9)	
Bachelors or higher	50 (9.7)	9 (5.5)	14 (14.9)	
Missing*	8 (1.6)	1 (0.6)	1 (1.6)	
Health Insurance				<.0001
Insured	106 (20.6)	18 (11.0)	33 (35.1)	
Uninsured	407 (79.2)	146 (89.0)	61 (64.9)	
Missing*	1 (0.2)	0 (0)	0 (0)	
US Born				<.0001
Yes	104 (20.2)	0 (0)	52 (55.3)	
No	409 (79.6)	164 (100.0)	42 (44.7)	
Missing*	1 (.2)	0 (0)	0 (0)	
Years in US				<.0001
< 10	125 (24.3)	60 (36.6)	4 (4.3)	
11–20 years	115 (22.4)	48 (29.3)	12 (12.8)	
20+	256 (49.8)	50 (30.5)	78 (82.9)	
Missing*	18 (3.5)	6 (3.6)	0 (0)	
English Fluency				<.0001
Poor of Fair	363 (70.6)	154 (93.9)	31 (33.0)	
Good or Excellent	141 (27.4)	8 (4.3)	62 (65.9)	
Missing*	10 (2.0)	3 (1.8)	1 (1.1)	
Spanish Language Preference				<.0001
Yes	446 (86.8)	164 (100.0)	70 (74.5)	
No	64 (12.4)	0 (0)	21 (22.3)	
Missing*	4 (0.8)	0 (0)	3 (3.2)	
BMI				0.0460
Underweight	3 (0.6)	0 (0)	2 (2.1)	
Normal	84 (16.3)	21 (12.8)	21 (22.3)	
Overweight	171 (33.3)	61 (37.2)	28 (29.8)	
Obese	250 (48.6)	82 (50.0)	43 (45.7)	
Missing*	6 (1.2)	0 (0)	0 (0)	
	**Mean (SD**	**Mean (SD)**	**Mean (SD)**	
Age	45.4 (13.0)	47.0 (10.9)	42.7 (15.7)	
Body Mass Index	30.5 (5.6)	31.01 (5.7)	29.8 (6.1)	
Body Fat Percentage	42.0 (10.1)	43.3 (8.1)	39.7 (10.8)	

*Missing completely at random if participant declined to answer or CHW skipped question

Acculturation was significantly correlated with BFP (*r* = − 0.17, *p* < .01) but not BMI, (*r* = − 0.06, *p* = .17). Socioeconomic status (SES) was also significantly correlated with both BFP (*r* = − 0.18, *p* < .01) and BMI (*r* = − 0.13, *p* < .01). Age was also significantly correlated with both BFP (*r* = 0.13, *p* = 0.01) and BMI (*r* = 0.12, *p* < 0.01). Gender, being male, was significantly correlated with BFP (*r* = − 0.58, *p* < .01) but not BMI (*r* = − 0.08, *p* = 0.09). All Pearson correlations are presented in Table 2.

**Table 2:** Pearson’s correlation coefficients

	BMI	BFP	Acculturation	SES	Age
BFP	.70*	–			
Acculturation	−.06	−.17*	–		
SES	−.13*	−.18*	.43*	–	
Age	.12*	.13*	−.23*	−.38*	–
Gender (male)	−.08	−.58*	.18*	.10*	−.02

Abbreviations: BMI, body mass index; BFP, body fat percentage; SES, socioeconomic status

*p < .05

The first hypothesis, that acculturation predicted higher baseline body composition, was tested using multivariate regression models predicting BMI and BFP, presented in Table 3. There were no significant interactions between covariates and acculturation, thus no interaction terms were included in the analysis. Several influential observations were identified, only slightly increasing parameter estimates, but removing them did not substantially alter findings and we concluded the observations were true possibilities, keeping them in the analysis. The tests of regression assumptions were satisfactory except for the Shapiro-Wilk’s W, which tests for normality of residuals [35]. Given that regression estimates are robust to this assumption in larger samples, we did not perform variable transformations [36].

**Table 3:** Regression models predicting BMI and BFP at baseline

	Body Mass Index (***n*** = 508)		Body Fat Percentage (***n*** = 465)^**1**^	
Independent Variables	B	95% CI	B	95% CI
Intercept	29.09*	(27.20, 30.97)	41.15*	(38.38, 43.92)
Acculturation	0.09	(−0.79, 0.98)	0.13	(−1.18, 1.43)
Socioeconomic Status	−0.70	(−1.47, 0.06)	− 1.32*	(−2.41, − 0.23)
Age	0.04	(−0.00, 0.07)	0.06*	(0.00, 0.12)
Gender	−1.06	(−2.42, 0.3)	−14.99*	(− 16.97, − 13.02)

^1^BFP was sometimes missing due to technical challenges with the biometric impedance scale, which consistently provided weight readings but was sometimes unable to estimate BFP

*p < .05

Our first hypothesis was not supported, as acculturation was not a significant predictor of BMI (*b* = 0.09, *p* = 0.83) or BFP (*b* = 0.13, *p* = 0.85). Although not statistically significant, the high acculturated group had lower BMI and BFP at baseline, 29.82 and 39.79%, respectively, compared to the less acculturated group, 31.02 and 43.31%, respectively.

For participants with 12-month follow-up measures (*n* = 258), BMI increased by 0.01 units, from 30.58 to 30.59, which is not statistically significant (t (257) = − 0.14, *p* = 0.89). However, BFP significantly decreased by a percentage of 0.71, from 42.03 to 41.32% (t (257) = 2.47, *p* = 0.01).

The multivariate regressions predicting BMI and BFP at the 12-month follow-up are presented in Table 4. Only participants with both body composition measurements at 12-month follow-up were included in the follow-up regression analyses (*n* = 258). Acculturation was a significant predictor for both BMI (*b* = 0.30, *p* = 0.03) and BFP (*b* = 1.33, *p* = 0.01). That is, for every standard deviation unit increase in acculturation, BMI increased by 0.30 and BFP increased by 1.33%, while controlling for socioeconomic status, gender, age, and body composition baseline levels. Follow-up analyses used a mean split to create a high acculturation and a low acculturation group.

**Table 4:** Regression models predicting BMI and BFP at 12-month follow-up, controlling for baseline measures

	Body Mass Index (***n*** = 258)		Body Fat Percentage (***n*** = 258)	
Independent Variables	B	95% CI	B	95% CI
Intercept	1.59	(0.65, 2.52)	6.95*	(3.44, 10.46)
Baseline Measure	0.97*	(0.94, 0.99)	0.86*	(0.79, 0.92)
Acculturation	0.30*	(0.03, 0.58)	1.33*	(0.35, 2.31)
Socioeconomic Status	0.08	(−0.22, 0.002)	0.05	(−0.78, 0.89)
Age	−0.01	(−0.60, 0.32)	− 0.04	(− 0.08, 0.01)
Gender	− 0.14	(− 0.32, 0.52)	−1.36	(−3.22, 0.51)

*p < .05

**Fig. 1:**
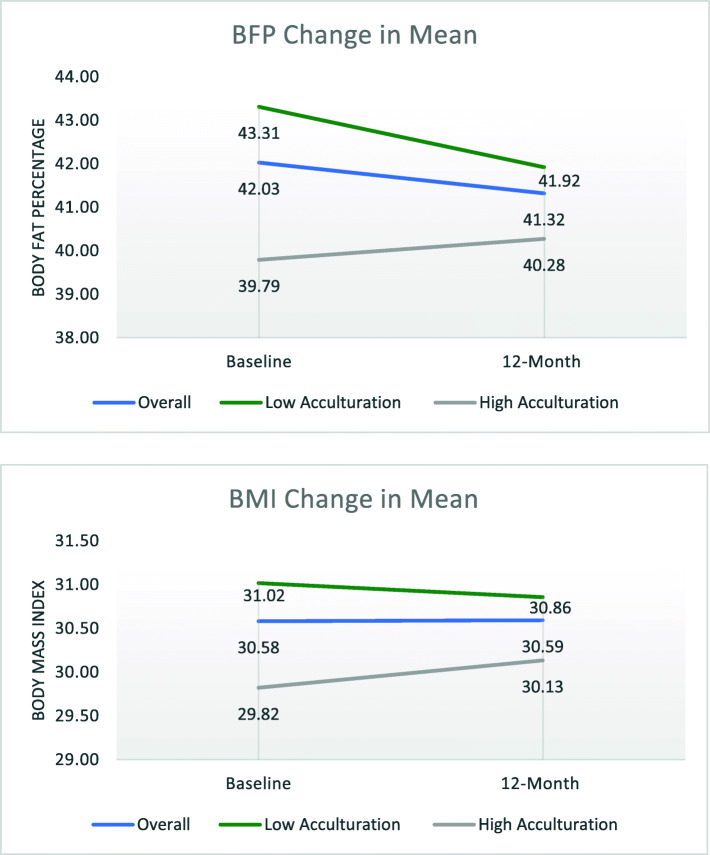
Graphic illustration of the changes in means over time for both Body Mass Index and Body Fat Percentage overall and by level of acculturation for all participants with 12-month follow-up measurements (*n* = 258 overall, *n* = 164 for low acculturation, *n* = 94 for high acculturation)

Figure 1 illustrates the changes in means for both body composition measures over time, overall (blue line) and by low and high level of acculturation (green and grey lines, respectively). The high acculturation group (*n* = 94) had a lower mean BMI of 29.82 at baseline, which increased to 30.13 at follow-up. The low acculturation group (*n* = 164) had a mean BMI of 31.02 at baseline, which decreased to 30.86 at follow-up. The same trend exists for BFP, where the high acculturation group had a mean BFP of 39.79% and increased to 40.28% while the low acculturation group decreased from 43.31 to 41.92%. That is, the low acculturation group improved (decreased) in body composition measures and the high acculturation group did not improve in either measure. The average weight loss overall for Healthy Fit participants was only 0.04 pounds, which is not clinically significant weight loss. By acculturation, the low acculturation group experienced an average decrease of 0.39 pounds while the high acculturation group experienced an average increase of 0.81 pounds.

We also tested the significance of differences between acculturation groups using independent sample t-tests. The t-tests compared the high and low acculturation groups on BMI and BFP change scores. Change in BMI from baseline to 12-month follow-up was 0.31 units in the high acculturation group and  -0.16 units in the low acculturation group, for a difference of 0.47 between the two groups (95% CI: − 0.82, − 0.13, *t* = − 2.73, df = 256, *p* < 0.05). Similarly, the change in BFP was 0.49% in the high acculturation group and  -1.39% in the low acculturation group, for a difference of 1.87% between the two groups (95% CI: − 3.02, − 0.71, *t* = − 3.2, df = 256, *p* < 0.05).

## Discussion

This study presented the outcomes of the Healthy Fit program among a sample of primarily low-income Hispanics living on the US-Mexico border, specifically analyzing the relation between acculturation and body composition changes over time. Although acculturation was not a significant predictor of body composition at baseline, it was a significant predictor of change from baseline to follow-up.

Contrary to the first hypothesis that acculturation would be associated with overweight or obesity, the more acculturated participants measured lower BMI and BFP compared to less acculturated participants at baseline. This result may be in part to the correlation between acculturation and SES since SES is a protective factor against overweight/obesity. In particular, people with higher income are more likely to afford healthy food options and live-in neighborhoods where they can safely and regularly exercise. Additionally, the average length of residence in the US for this sample is 21 years, which far exceeds the 10-year threshold noted in previous research where significant weight gain was seen [16].

Although BFP did decline over time, there were no reductions in BMI in the overall sample. However, BMI remaining stable over time is also a positive outcome, considering that some weight gain is normal with age. Furthermore, BFP is a stronger indicator for overweight/obesity and disease risk since it can differentiate between lean mass and fat mass [36, 37]. Although Healthy Fit had a small effect on BFP, long-term implementation could lead to more substantial health benefits. For obese patients, losing a minimum of 5% of their body weight lowers risk for diabetes, cardiovascular disease, and other obesity-related conditions [38–40]. Although Healthy Fit participants did not, on average, experience clinically significant weight loss after 12-months, effects might be compounded over time if the low-cost, light touch intervention was sustained.

Regarding changes by level of acculturation, the low acculturated group experienced significant reductions in both BMI and BFP not seen among the high acculturated group. As expected, the culturally tailored educational material and delivery by low acculturated CHWs increased effectiveness allowing small but significant changes among the low acculturated group. However, the absence of weight loss among the more acculturated participants highlights the need for additional cultural tailoring. Tremendous variability exists among Hispanics, and this study suggests acculturation status captures important differences within the heterogeneous Hispanic population. Findings also suggest it may be important to intervene early with immigrants before individuals adopt behaviors and attitudes that negatively affect health behaviors and outcomes. The high acculturated group may have a different perception of healthy weight standards or image, varying access to healthy or processed foods, or other health behaviors which may have impacted the effectiveness of the program. It may also be that the low acculturated group has not transitioned from their consumption of healthier foods to processed fatty foods, as theorized by the nutritional transition theory [16]. Additional research is needed to further explore why the high acculturation group did not benefit from the program.

The study findings have important implications for the development and implementation of culturally appropriate weight loss interventions which may need to vary depending on level of acculturation. This study contributes to the existing literature on the impact of acculturation on health outcomes specifically for US-Mexico border populations by providing an understanding of the factors that predict obesity and overweight among a US-Mexico border population. The analysis between two acculturation groups, high and low, also fills a gap in knowledge regarding intervention development and implementation by stressing the importance of culturally appropriate delivery.

### Strengths and limitations

A major strength of this study is the large sample size providing adequate power in detecting significant differences. Further, we succeeded in reaching a vulnerable population facing substantial health disparities and tracking their health outcomes over time. However, the generalizability of the results may be limited to Hispanic groups living on the border and may not apply directly to other ethnic groups or regions.

The culturally tailored educational material and intervention delivery by CHWs may have increased effectiveness of the intervention allowing small but significant changes among the low acculturation group. The ability of CHWs to provide culturally appropriate education to this group by means of communication and use of fotonovelas is one of the strengths of the Healthy Fit intervention. In addition, since CHWs are widely used in the US-Mexico border region for educational programs, they may be generally perceived as trustworthy, fostering program effectiveness.

Further research is needed to fully understand the role of acculturation on body composition and why the intervention was more successful among less acculturated participants. Additional variables such as body perception, ideal body image, perceived benefits and barriers of physical activity and healthy eating, and country of origin could be analyzed to understand how they impact body composition outcomes between the acculturation groups. Future studies should also aim to include more men, as this study sample primarily consists of women. To fully understand this relationship between acculturation and body composition, researchers should consider an ecological perspective in analyzing the impact of acculturation on health behaviors by probing cultural beliefs, social norms, and support networks of the individual and broader factors such as the social and political climate of the community, access to resources, and the physical environment.

## Conclusion

This study contributes to research and practice by highlighting the importance of acculturation when developing interventions for Hispanic populations. Specifically, acculturation was a significant predictor of body composition changes over time following a weight-loss intervention targeting Hispanics living in the US-Mexico border region. Healthy Fit’s culturally tailored approach was successful for less acculturated individuals. As such, level of acculturation should be considered in the development and implementation of obesity prevention interventions. This light-touch intervention could be implemented on a broader scale to address obesity disparities among similar vulnerable communities. More research is needed to identify effective strategies for more acculturated Hispanic populations.

## Data Availability

The dataset analyzed during the current study are available from the corresponding author on reasonable request.

## Abbreviations

BFP: Body Fat Percentage
BMI: Body Mass Index
CHW: Community Health Worker
HIE: Healthy Immigrant Effect
US: United States of America
